# Evaluation of dental students’ awareness about intraoral scanners

**DOI:** 10.1371/journal.pone.0335940

**Published:** 2025-10-30

**Authors:** Berrak Çakmak, Ebubekir Yıldız, Tuba Tortop

**Affiliations:** 1 Department of Orthodontics, Faculty of Dentistry, Gazi University, Ankara, Turkey; 2 Faculty of Dentistry, Gazi University, Ankara, Turkey; International Medical University, MALAYSIA

## Abstract

**Background:**

There are a few studies evaluating dental students’ knowledge and awareness of intraoral scanners. This study aims to evaluate and compare the knowledge and awareness of levels 3^rd^, 4^th,^ and 5^th^-grade dental students regarding intraoral scanners and their use in orthodontics.

**Methods:**

A survey adapted from similar studies was administered to a total of 278 volunteer undergraduate students [comprising 3^rd^ (n = 94), 4^th^ (n = 88), and 5^th-^grade (n = 96)] to assess their awareness of intraoral scanners. Descriptive statistics, including frequencies and percentages, were used to analyze the variables within the scope of the study. The Fisher–Freeman–Halton exact test was employed to compare responses across different academic years. A p-value of <0.05 was considered statistically significant.

**Results:**

Of the participants, 33.81% were 3^rd^-grade, 31.65% were 4^th^-grade, and 34.53% were 5^th^-grade dental students. There was no significant difference in the distribution of participants across academic grades (p > 0.05). Among the students, 96.81% of the 3^rd^-grade, 89.77% of the 4^th^-grade, and 83.33% of the 5^th^-grade students reported that they had never used an intraoral scanner (p < 0,05). A higher proportion of 3^rd^-grade students (71.28%) believed that IOSs were used in the treatment of skeletal Class II malocclusions and the fabrication of maxillary expansion appliances, compared to the other grade groups. In contrast, 5^th^-grade students more frequently associated IOS usage with indirect bonding procedures (63.54%) (p < 0.05).

**Conclusion:**

Students in the 4^th^ and 5^th^ grades demonstrated a greater level of knowledge compared to 3^rd^-grade students. It is recommended that practical training opportunities be expanded and the dental curriculum be revised accordingly to support hands-on experience with intraoral scanners.

## Introduction

Digital dentistry is continuously enhancing the speed, accuracy, and comfort of patient treatment procedures [1–3]. In this context, intraoral scanners play an important role in the application of digital technologies. Intraoral scanners (IOS) are devices used to generate high-resolution, three-dimensional (3D) digital models of teeth and surrounding oral tissues [4]. Compared to conventional impression techniques, IOS offer several advantages, including reduced chairside time [2–7], greater precision in treatment planning [2–5,7], increased patient comfort [2–5,7], easy repeatability [3–5,7], elimination of the need for physical storage, reduced risk of impression material distortion, and removal of the requirement for plaster model fabrication [3–5].

In recent years, the integration of digital technologies into dental education programs has gained increasing importance in order to equip students with comprehensive knowledge and hands-on experience in contemporary treatment methods. However, compared to traditional impression techniques, training in dental school curricula generally allocates less time to training for IOS, potentially due to limitations in time or equipment availability [8–10]. This may limit students’ ability to effectively use digital technologies in clinical practice after graduation. Marti et al. [11] reported that dental students have high expectations of IOS and prefer them as the primary impression techniques in their future professional practice, and suggested that curricula should be adapted accordingly. Similarly, Schott et al. [12] evaluated traditional and digital impression techniques from the student perspective, and concluded that students preferred the digital impression techniques, emphasizing the need for IOS hands-on training to be incorporated into undergraduate dental education.

Understanding students’ knowledge and usage experiences of IOS is important to determine the necessary steps for the integration of the curriculum into digital technologies [10,13,14]. Although there are many studies [5,6,10,11,15,16] evaluating IOS awareness, they either evaluated the preferences of dentists and dental students or assessed only graduate students and a single undergraduate class. A limited number of studies [17–19] have conducted grade-based comparative evaluations related to IOS. While Güntekin et al. [17] investigated the knowledge level of dental students mainly about the applications of IOS in prosthetic treatments and implantology, Sharab et al. [18] evaluated their perceptions and awareness about IOS. In the literature, no study focusing on the awareness of dental students regarding the use of IOS in orthodontics was found. However, with the increasing prevalence of clear aligner therapy and digital appliances in orthodontics, the use of IOS is also becoming more widespread in this field. In this context, this study aimed to examine and compare the knowledge levels of dental students (3^rd^, 4^th^, and 5^th^-grade) regarding IOS and their awareness of their use in the field of orthodontics. The hypothesis of the study was that 5^th^-grade dental students had more knowledge and awareness about IOS than students in other grades.

## Materials and methods

Ethics commission approval for the study was given by Gazi University Ethics Commission (11.06.2024; Ethics Committee Decision No: 2024/1006). The study was carried out in line with the Declaration of Helsinki, between 20 June 2024 and 20 July 2024. The study was also performed following the STROBE (Strengthening the Reporting of Observational Studies in Epidemiology) guidelines [20]. All participants signed an informed consent form before their inclusion in the study, indicating their voluntary participation. This study was designed as a cross-sectional descriptive survey. The inclusion criterion for the study was being a 3^rd^, 4^th^, or 5^th^-grade student at Gazi University, Faculty of Dentistry. Participants who did not sign the informed consent form, were repeating the academic year, did not complete the survey, or provided duplicate responses were excluded from the study. A pilot test conducted with 30 students at 2-week intervals showed that no changes were needed to the survey. After determining that the survey was reliable, the main study began.

**Fig 1:**
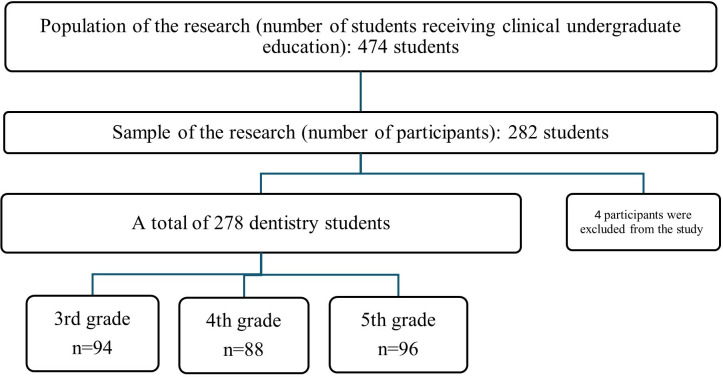
Flowchart of the sample selection process.

The population of the research consisted of all dental students receiving clinical undergraduate education at Gazi University Faculty of Dentistry (a total of 474 dental students in the 3^rd^-grade (164 students), 4^th^-grade (142 students), and 5^th^-grade (168 students). Since a minimum of 50% availability was targeted in the sample, the study was planned to be conducted with a minimum of 237 participants. The survey was conducted in a lecture hall setting, and the surveys were distributed to the students and collected after they completed them anonymously during the same session (S1 File). Participation was voluntary. The research sample consisted of 282 student participants. The age range was 20–26 years old. Four participants were excluded from the study because they did not provide a response. A total of 278 dental students (3^rd^-grade (n = 94), 4^th^-grade (n = 88), and 5^th^-grade (n = 96)) were included in the study (Fig 1).

The questions in the survey were inspired by similar studies [9,10] conducted to measure dental students’ awareness of IOS. The survey included a total of 13 questions, eight questions with yes/no answers, and five questions with multiple-choice answers (Table 1). The language of the survey was Turkish. The answering time of the survey was determined to be approximately 5 minutes.

**Table 1:** Survey questions and comparison of survey responses according to dental students’ grades (n: number, %: percentage).

Survey Questions	3^rd^ grade n (%)	4^th^ grade n (%)	5^th^ grade n (%)	p
1. What grade are you studying in?				
3^rd^ grade	94 (33,81%)^a^			0.831
4^th^ grade		88 (31,65%)^a^		
5^th^ grade			96 (34,53%)^a^	
2. Do you know what an IOS is?				
Yes	89 (94,68%)^a^	84 (95,45%)^a^	96 (100%)^a^	0,057
No	5 (5,32%)^a^	4 (4,55%)^a^	0 (0%)^a^	
3. Do you know how the IOS works?				
Yes	57 (60,64%)^a^	65 (73,86%)^a^	68 (70,83%)^a^	0,133
No	37 (39,36%)^a^	23 (26,14%)^a^	28 (29,17%)^a^	
4. Have you used the IOS?				
Yes	3 (3,19%)^a^	9 (10,23%)^ab^	16 (16,67%)^b^	0,006*
No	91 (96,81%)^a^	79(89,77%)^ab^	80 (83,33%)^b^	
5. Would you like to experience using an IOS?				
Yes	89 (94,68%)^a^	81 (92,05%)^a^	88 (91,67%)^a^	0,706
No	5 (5,32%)^a^	7 (7,95%)^a^	8 (8,33%)^a^	
6. Have you watched any educational videos or attended seminars about the IOS outside of faculty?				
Yes	22 (23,40%)^a^	29 (32,95%)^a^	24 (25%)^a^	0,403
No	72 (76,60%)^a^	59 (67,05%)^a^	72 (75%)^a^	
7. Do you think the IOS will contribute to your professional development?				
Yes	91 (96,81%)^a^	84 (95,45%)^a^	94 (97,92%)^a^	0,581
No	3 (3,19%)^a^	4 (4,55%)^a^	2 (2,08%)^a^	
8. Do IOS offer dentists a more convenient and faster treatment option?				
Yes	94 (100%)^a^	88 (100%)^a^	91 (94,79%)^b^	0,012*
No	0 (0%)^a^	0 (0%)^a^	5 (5,21%)^b^	
9. Which techniques do you think are advantageous in dental treatment planning?				
IOS	92 (97,87%)^a^	84 (95,45%)^a^	91 (94,79%)^a^	0,549
Making a traditional impression	2 (2,13%)^a^	4 (4,55%)^a^	5 (5,21%)^a^	
10. What are the advantages of IOS for patients?				
Cheap cost	15 (15,96%)^a^	17 (19,32%)^a^	22 (22,92%)^a^	0,489
Patient comfort	82 (87,23%)^a^	80 (90,91%)^a^	79 (82,29%)^a^	0,214
Quick treatment planning	77 (81,91%)^a^	72 (81,82%)^a^	68 (70,83%)^a^	0,092
11. What method does IOS use to create a digital model?				
Sound wave	10 (10,64%)^a^	10 (11,36%)^a^	12 (12,50%)^a^	0,945
Magnetic Resonance	20 (21,28%)^a^	20 (22,73%)^a^	17 (17,71%)^a^	0,684
Optical Imaging	77 (81,91%)^a^	75 (85,23%)^a^	78 (81,25%)^a^	0,781
12. In which treatment methods do you think IOS are used?				
In temporomandibular joint disorders	21 (22,34%)^a^	25 (28,41%)^a^	25 (26,04%)^a^	0,637
In the treatment of skeletal class 2 cases	67 (71,28%)^a^	46 (52,27%)^b^	57 (59,38%)^b^	0,018*
To take precise impressions in implant surgery	70 (74,47%)^a^	54 (61,36%)^a^	65 (67,71%)^a^	0,168
In the design and production of prostheses	83 (88,30%)^a^	77 (87,50%)^a^	81 (84,38%)^a^	0,736
Aligner treatment in orthodontics	84 (89,36%)^a^	71 (80,68%)^a^	80 (83,33%)^a^	0,241
13. What are the uses of IOS in the field of orthodontics?				
Prepare a model	86 (91,49%)^a^	75 (85,23%)^a^	76 (79,17%)^a^	0,053
In the construction of maxillary expansion appliances	67 (71,28%)^a^	40 (45,45%)^b^	48 (50,00%)^b^	<0,001*
Preparation of brackets for indirect bonding	42 (44,68%)^a^	36 (40,91%)^a^	61 (63,54%)^b^	0,004*
In aligner treatment	85 (90,43%)^a^	70 (79,55%)^a^	87 (90,63%)^a^	0,052
In the construction of myofunctional appliances	51 (54,26%)^a^	40 (45,45%)^a^	45 (46,88%)^a^	0,442

* statistically significant (p < 0,05), (Fisher-Freeman-Halton Exact tests).

Data presented as number (percentage) (n (%)).

a-b There is no difference between the rates of grades with the same letter.

### Statistical analysis

Statistical analyses were performed using SPSS 29 (IBM SPSS Statistics, SPSS 29.0 version) statistical software. The intraclass correlation coefficient (ICC) was used to calculate reliability in the pilot study. Frequency and percentage were used in the descriptive statistics of the variables examined within the scope of the research. Cronbach’s alpha coefficient was used to assess reliability. The Fisher-Freeman-Halton Exact test was used to investigate the relationship between categorical data. Post-hoc tests were used for multiple comparisons between grades. A p-value of <0.05 was considered statistically significant.

## Results

The range of 0.77 to 1 in ICC values indicated good reliability. Cronbach’s alpha value of 0.79 indicates good intra-rater reliability. For the study’s power, a minimum target of 50% accessibility (n = 237) was set for the population, and the actual accessibility achieved was 58.64% (n = 278). 33.81% of the participants were in their third, 31.65% in their fourth, and 34.53% in their fifth academic year (Table 1). The distribution of participants among the groups did not show any significant differences. (p > 0.05).

It was observed that 96.81% of 3^rd^-grade, 89.77% of 4^th^-grade, and 83.33% of 5^th^-grade students had not used iOS. (p < 0.05). There was a difference in IOS usage rates between the 3^rd^-grade and 5^th^-grade students. All 3^rd^ and 4^th^-grade students, along with 94.79% of the 5^th^-grade students, believed that IOS offered dentists a more comfortable and faster treatment option. The differences between grades were statistically significant (p < 0.05).

**Fig 2:**
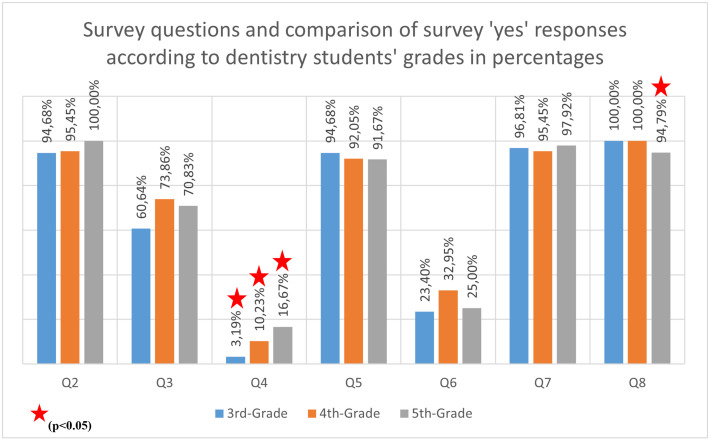
Survey questions (Q2-Q8) and percentage distributions according to grades.

**Fig 3:**
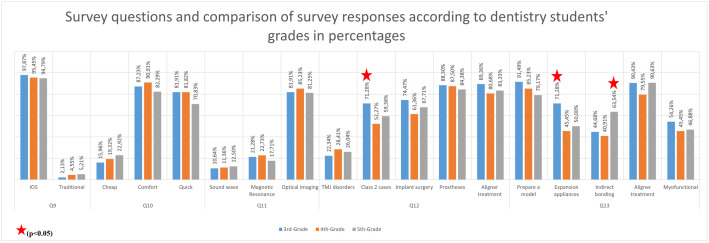
Survey questions (Q9-Q13) and percentage distributions according to grades.

The 3^rd^-grade students believed that IOS was utilized in the treatment of skeletal Class 2 cases more frequently (71.28%) than the other grades. In comparison, the 4^th^-grade students estimated it at 52.27%, and the 5^th^-grade students at 59.38% (p < 0.05). Additionally, the 3^rd^-grade students also thought that IOS was employed in the fabrication of maxillary expansion appliances at a higher rate (71.28%) than the other grades, with the 4^th^-grade students at 45.45% and the 5^th^-grade students at 50% (p < 0.001). The 5^th^-grade students thought that IOS was used in the preparation of brackets for indirect bonding at a higher rate (63.54%) than the other grades (^3rd^-grade students: 44.68%, 5^th^-grade students: 40.91%) (p < 0.05). (S1 Table in S1 Appendix). The findings were visualized graphically in Figs 2 and 3.

## Discussion

As digital technologies increasingly permeate the field of modern dentistry, IOS have emerged as indispensable tools within the digital workflow, playing a pivotal role in enhancing clinical precision and efficiency. Understanding how dental students perceive and comprehend these tools is essential, given that they represent the future workforce responsible for implementing such technologies in routine practice. Therefore, evaluating the grade among students at different stages of education can provide valuable insights into the effectiveness of current curricula and highlight potential areas for enhancement in digital dentistry training. The present study was conducted to comparatively assess the knowledge and awareness levels of dental students in the 3^rd^, 4^th^, and 5^th^ grades of their education regarding IOS, particularly in the context of clinical and orthodontic training.

In the curriculum of Gazi University Faculty of Dentistry, IOS and traditional impression techniques are included for all three grades (3^rd^, 4^th^, and 5th grades). However, the depth and emphasis of theoretical instruction vary across these grades. In the 3rd grade, IOS is introduced in the preparedness ‘Orthodontics’ and ‘Prosthodontics’ theory classes. Students are taught basic concepts such as the working principles of IOS, comparison with conventional impression techniques, and an overview of digital workflow in dentistry. At this stage, the focus is primarily conceptual. In the 4th grade, the content expands within classes like ‘Orthodontics’ and ‘Digital Dentistry’. Students learn more advanced topics such as scanning strategies, common scanning errors, advantages and limitations of IOS in different clinical situations, and the integration of IOS with CAD/CAM systems. In the 5th grade, the emphasis shifts toward clinical application. Through the ‘Dentist-Orthodontist Collaboration’ class and internship, students observe or participate (by individual request) in patient treatments involving IOS. Theoretical instruction includes case-based discussions, digital treatment planning, and interpretation of scan data.

Traditional impression techniques are introduced through peer practice in the second semester of the 3^rd^ grade and are further reinforced through clinical application on patients during the internship periods in the 4^th^ and 5^th^ grades. IOS is taught only in theory classes, and an obligatory practical application cannot be made due to the inadequacy of IOS in the faculty and time constraints. Students can observe IOS in line with their individual requests during clinical observation and internship periods. The clinical observation period in the 3^rd^ grade is only 6 days, while the 4^th^ and 5^th^ grade internships last a minimum of 20 days.

Clinical observation and orthodontic education at our university begin in the 3rd grade. Considering that exposure to the material rather than just theoretical education increases awareness, grades were created starting from the 3^rd^-grade students, and the 1^st^ and 2^nd^ grades were not included in this study.

This study showed that the majority of dental students know what IOS is and how it works. Similarly, Ketharinath et al. [19] reported that IOS awareness among dental students was over 80%. The way dental students perceive IOS is largely influenced by their training experiences. Zoidis et al. [21] highlight that students exposed to comprehensive training programs view IOS as valuable assets for their future practice.

This study indicated that overall, very few dental students had experience using IOS. Similar to our findings, Güntekin et al. [17] reported that the IOS usage rate of dental students was 12.4%, and Merchant et al. [22] reported that 70% of dental students had no experience using IOS. In this study, the higher rate (16,67%) of IOS use by 5^th^-grade students compared to other grades was probably due to their advancement in clinical practice. The difference in experience in using IOS shows how important clinical practice is in dental education. Students in the early years of dental education should be offered more clinical practice opportunities, increased access to digital dentistry equipment, and greater interaction with patients. Because digital dentistry is becoming increasingly widespread, and devices such as IOS are becoming an integral part of clinical practice. Students who are familiar with digital technologies in the first grade can use these systems more quickly and effectively. The fact that only 25% of the students were exposed to IOS-related training outside the faculty underlined the importance of the training provided in the faculty. Corne et al. [23] reported that dental students using IOS without teacher support were unable to improve their performance and self-assessment abilities. Similarly, Christopoulou et al. [24] reported in their systematic review that the accuracy and reproducibility of intraoral scanning require experience and good clinical skills to overcome the limitations. In addition, the majority of the students believe that using IOS will contribute to their professional development. Therefore, faculty training should not be limited to theoretical knowledge, and IOS practical application should also be included in the curriculum. Similar to our findings, Lam et al. [10] reported that dental students have a positive view of IOS, and with more practice and clinical exposure to IOS, more students may prefer IOS. Alqahtani et al. [16] reported that while dental students generally had a positive attitude toward IOS, they lacked sufficient knowledge and practical experience, emphasizing the need for enhanced educational initiatives to support the broader adoption of IOS in dental practice. Limited exposure may lead to skepticism or resistance to the adoption of these technologies.

In this study, dental students found IOS more comfortable, faster, but more costly than the traditional impression techniques. Similarly, Lam et al. [10], Scott et al. [12], Ketharinath et al. [19] and, Alfallaj et al. [25] reported that dental students found IOS to be faster and more effective than traditional impression techniques. A study by Ahmet et al. [26] reported that 71% of dental students had a positive experience with IOS and were likely to adopt this technology in the future due to their perception of time savings compared to traditional impression techniques. Similar to the findings of many studies [2,7,21], dental students consider IOS to be more advantageous than traditional impression making. However, Lam et al. [10] reported that despite the existing advantages of IOS, many dental students prefer making traditional impressions, which is more efficient for them.

All grades reported similar perceptions regarding the use of IOS in temporomandibular joint disorders, precision impression for implant surgery, prosthesis design and fabrication, and aligner therapy. However, 3^rd^-grade students perceived a higher rate of IOS utilization in the treatment of skeletal Class II malocclusions compared to students in other years. This divergence may be attributed to their limited and superficial understanding of the subject matter, suggesting that their responses may not reflect informed or conscious clinical reasoning.

When the field of orthodontics is specifically considered, students across all academic years reported similar perceptions regarding the use of IOS in model fabrication, clear aligner therapy, and the production of myofunctional appliances. However, 3^rd^-grade students indicated a higher perceived usage of IOS in the fabrication of maxillary expansion appliances. This finding may be attributed to their exposure to such appliances during clinical observation sessions in the third year. Additionally, 5^th^-grade students reported a higher perceived use of IOS in the preparation of brackets for indirect bonding compared to students in other grades. This discrepancy is likely due to the limited exposure to or knowledge of indirect bonding protocols among students. Based on the findings, the hypothesis of the study was accepted.

The primary limitation of this study is that it was conducted within a single institution. Furthermore, some students may have conflated the concepts of IOS and three-dimensional printer, which could have influenced their responses. The findings indicated that while students possessed a relatively high level of theoretical knowledge about IOSs, their hands-on experience was notably insufficient. Although IOSs are utilized in clinical settings, students often do not have the opportunity to operate them directly. This lack of practical exposure may influence their perceptions of IOS technology. Nonetheless, the students demonstrated a strong willingness to enhance their knowledge and skills in this area.

Integrating both theoretical and practical training on IOSs into earlier phases of clinical education (preferably before the 4th grade) would likely improve students’ competence, particularly in orthodontic applications. Such early exposure would help establish a solid foundation by the time students reach their final year, thereby enabling them to transition into professional practice with greater confidence and proficiency.

Given the increasing adoption of IOS in dental practice, further studies are warranted to evaluate its optimal integration into dental curricula. Although the global implementation of IOS remains uneven, potentially due to resource limitations, its future significance in dental education and practice is evident. As the use of IOS expands and becomes more embedded in educational frameworks, continuous updates in research and curricular content will be necessary. Additionally, since this study was limited to a single faculty, conducting comparative studies across multiple institutions and countries would enhance the generalizability of the findings.

## Conclusion

- 4^th^ and 5^th^-grade dental students demonstrated a greater degree of familiarity with IOS compared to 3^rd^-grade dental students.
- Dental students believed that IOS technology would contribute positively to their professional development and offer significant advantages for patient care.
- Expanding educational resources and providing more hands-on training opportunities may further enhance students’ knowledge and engagement with this technology.
- In the field of orthodontics, the increasing use of clear aligner systems, digital model production, virtual treatment simulations, and CAD/CAM-supported appliance designs makes it essential for dental students to become familiar with these technologies before graduation. Therefore, dedicated IOS training should be incorporated into each relevant discipline within dental education.

## Supporting information

S1 File A survey applied to participants. (PDF)

S1 Fig Photo of all surveys administered to participants. (JPG)

S2 File Original data entry drafts. (PDF)

S1 Table Excel tables for statistics. (XLSX)
