# Supplementary material for: Evaluation of dental students’ awareness about intraoral scanners
Source: PLoS One. 2025 Oct 30;20(10):e0335940. doi: 10.1371/journal.pone.0335940 (PMC12574895; doi:10.1371/journal.pone.0335940)
Supplement: S1 File — (PDF) [file pone.0335940.s001.pdf]

Sayın katılımcı,

'Diş hekimliği öğrencilerinin ağız içi tarayıcılar hakkında farkındalığının değerlendirilmesi' başlıklı bu araştırma, Gazi Üniversitesi Diş Hekimliği Fakültesi Ortodonti Anabilim Dalı tarafından yapılmaktadır.

Araştırmanın amacı, 3. 4. Ve 5. sınıf diş hekimliği öğrencilerinin ağız içi tarayıcılar ile ilgili bilgi düzeylerinin incelenmesi ve karşılaştırma yapılarak ağız içi tarayıcılarla ilgili bilgi düzeyine klinik eğitimin etkisinin değerlendirilmesidir.

Araştırmaya katılmanız gönüllük esasına dayalıdır. Anket aracılığıyla elde edilecek bilgiler gizli kalacaktır ve sadece bilimsel amaçlar için kullanılacaktır. Anket kağıdına adınızı ve soyadınızı yazmayınız.

Çalışmamıza katıldığınız için teşekkür ederiz.

Çalışma ile ilgili herhangi bir sorunuz olduğunda aşağıdaki kişiler ile iletişim kurabilirsiniz:

Prof. Dr. Tuba Tortop

Öğr. Gör. Dr. Berrak Çakmak

Ebubekir YILDIZ

Gazi Üniversitesi Diş Hekimliği Fakültesi Ortodonti Anabilim Dalı

tubatortop@gazi.edu.tr

berrak@gazi.edu.tr

ebubekir.yildiz@gazi.edu.tr

Çalışmaya katılmayı kabul ediyorsanız aşağıdaki kutucuğu 'Çarpı (X)' ile işaretleyiniz.

☐ Kabul ediyorum

Scanned with CamScanner

Gazi Üniversitesi Diş Hekimliği Fakültesi öğrencisi olarak katılıyorsunuz. Lütfen aşağıdaki soruları yanıtlayınız.

1-) Hangi dönemde eğitim alıyorsunuz?

3. Dönem ☐

4. Dönem ☒

5. Dönem ☐

2-) Ağız içi tarayıcının ne olduğunu biliyor musunuz?

Evet ☒

Hayır ☐

3-) Ağız içi tarayıcının işleyişini biliyor musunuz?

Evet ☒

Hayır ☐

4-) Ağız içi tarayıcıyı hiç kullandınız mı?

Evet ☐

Hayır ☒

5-) Ağız içi tarayıcıyı kullanma deneyimi yaşamak ister misiniz?

Evet ☒

Hayır ☐

6-) Okul dışında ağız içi tarayıcı hakkında herhangi bir eğitim videosu izlediniz mi veya seminere katıldınız mı?

Evet ☐

Hayır ☒

7-) Ağız içi tarayıcının mesleki gelişimine katkıda bulunacağını düşünüyor musunuz?

Evet ☒

Hayır ☐

Scanned with CamScanner

8-) Diş Hekimliği tedavi planlamalarında hangi yöntemin avantajlı olduğunu düşünüyorsunuz?

Ağız İçi Tarayıcı ☒

Geleneksel Ölçü Alma Yöntemi ☐

9-) Ağız içi tarayıcılar diş hekimlerine daha uygun ve hızlı tedavi seçeneği sunar mı?

Evet ☒

Hayır ☐

10-) Ağız içi tarayıcıların hastalar için avantajları nelerdir?

Ucuz maliyet ☐

Hasta konforu ☒

Hızlı tedavi planlaması ☒

11-) Ağız içi tarayıcılar dijital model oluşturmak için hangi yöntemi kullanır?

Ses Dalgası ☐

Manyetik Rezonans ☒

Optik Görüntüleme ☐

12-) Aşağıdakilerden hangilerinde ağız içi tarayıcıların tedavi yöntemlerinde kullanıldığını düşünüyorsunuz?

TME bozukluklarında ☒

İskeletsel sınıf 2 vakaların tedavisinde ☐

İmplant cerrahisinde hassas ölçümler almak için ☐

Protezlerin tasarımı ve üretiminde ☐

Ortodontide şeffaf plak tedavisinde ☐

13-) Ağız içi tarayıcıların ortodonti alanında kullanım alanları aşağıdakilerden hangileridir?

Model hazırlamak ☒

Maksiller genişletme apareylerinin yapımında ☐

İndirek bonding için braketlerin hazırlanmasında ☐

Şeffaf plak tedavisinde ☐

Myofonksiyonel apareylerin yapımında ☐

Scanned with CamScanner
