# Supplementary figures and images for: Evaluation of dental students’ awareness about intraoral scanners
Source: PLoS One. 2025 Oct 30;20(10):e0335940. doi: 10.1371/journal.pone.0335940 (PMC12574895; doi:10.1371/journal.pone.0335940)

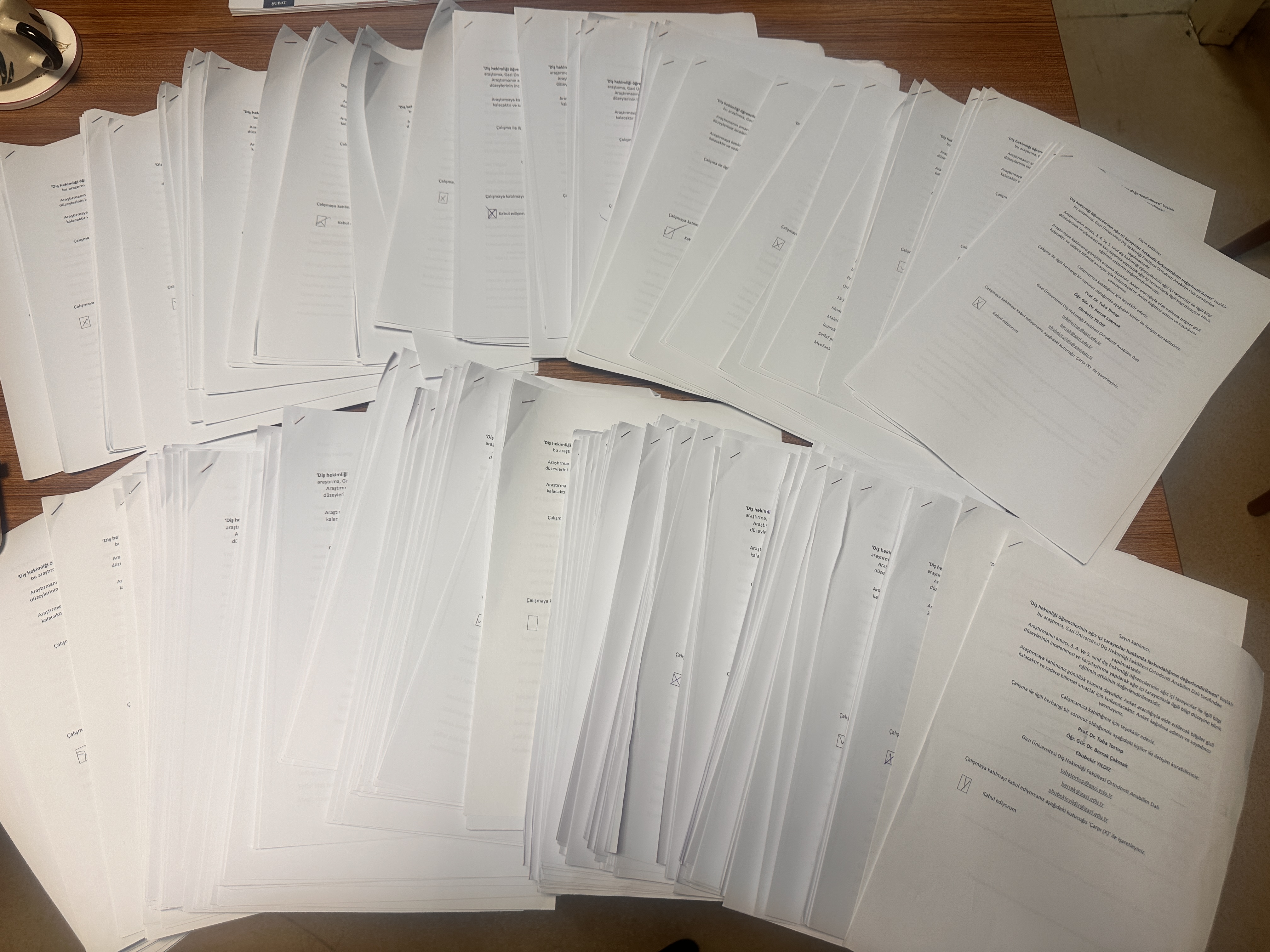

Supplement: S1 Fig — (JPG) [file pone.0335940.s002.JPG]
