# Supplementary material for: Evaluation of dental students’ awareness about intraoral scanners
Source: PLoS One. 2025 Oct 30;20(10):e0335940. doi: 10.1371/journal.pone.0335940 (PMC12574895; doi:10.1371/journal.pone.0335940)
Supplement: S2 File — (PDF) [file pone.0335940.s003.pdf]

## Dönem 4

1 kişi cevap vermemiş.

(2) 84 E, 4 H = 88
(3) 65 E, 23 H = 88
(4) 9 E, 79 H = 88
(5) 81 E, 7 H = 88
(6) 29 E, 59 H = 88
(7) 84 E, 4 H = 88

(8) Tarayıcı = 84, Geleneksel = 4
(9) 88 E, 0 H
(10) Ucuz = 17, Konfor = 80, Hızlı = 72
(11) Ses = 10, MR = 20, Optik = 75
(12) TME = 25, Cl 2 = 46, İmplant = 54
Protez = 77, Şeffaf plak = 71
(13) Model = 75, Max-geniş = 40, İndirekt = 36
Şeffaf plak = 70, miyofonks = 40

88 Kişi 1 kişi çalışma dışı = 89

---

## Dönem 3

2 kişi cevap vermemiş.

(2) 89 E, 5 H = 94
(3) 57 E, 37 H = 94
(4) 3 E, 91 H = 94
(5) 89 E, 5 H = 94
(6) 22 E, 72 H = 94
(7) 91 E, 3 H = 94

(8) Tarayıcı = 92, Geleneksel = 2
(9) 94 E, 0 H
(10) Ucuz = 15, Konfor = 82, Hızlı = 77
(11) Ses = 10, MR = 20, Optik = 77
(12) TME = 21, Cl 2 = 67, İmplant = 70
Protez = 83, Şeffaf plak = 84
(13) Model = 86, Max-geniş = 67, İndirekt = 42
Şeffaf plak = 85, miyofonks = 51

94 Kişi, 2 kişi çalışma dışı = 96

---

## Dönem 5

(2) 96 E, 0 H = 96
(3) 68 E, 28 H = 96
(4) 16 E, 80 H = 96
(5) 88 E, 8 H = 96
(6) 24 E, 72 H = 96
(7) 94 E, 2 H = 96

(8) Tarayıcı = 91, Geleneksel = 5
(9) 91 E, 5 H
(10) Ucuz = 22, Konfor = 79, Hızlı = 68
(11) Ses = 12, MR = 17, Optik = 78
(12) TME = 25, Cl 2 = 57, İmplant = 65
Protez = 81, Şeffaf plak = 80
(13) Model = 76, Max-geniş = 48, İndirekt = 61
Şeffaf plak = 87, Miyofonk = 45

96 kişi, 1 kişi çalışma dışı = 97

Scanned with CamScanner

[illegible]dnem 4

| | Evet | Hayır |
|---|---|---|
| 2 | 35 | 3 |
| 3 | 29 | 9 |
| 4 | 5 | 33 |
| 5 | 35 | 3 |
| 6 | 14 | 24 |
| 7 | 35 | 3 |

Scanned with CamScanner

8 — 4. Sınıf

| Tarayıcı | Geleneksel |
|---|---|
| 34 | 4 |

9

| Evet | Hayır |
|---|---|
| 38 | |

10

| Ucuz | Konfor | Hızlı Tedavi |
|---|---|---|
| 10 | 34 | 26 |

11

| Ses dalgası | M.R. | Optik |
|---|---|---|
| 7 | 14 | 27 |

12

| TME | İsk 2 | İmplant | Protez | Şeffaf Plak |
|---|---|---|---|---|
| 16 | 22 | 21 | 28 | 27 |

13

| Model h. | Max genz. | İndirekt B. | Şeffaf P. | Mayofonk. |
|---|---|---|---|---|
| 32 | 18 | 19 | 24 | 19 |

Dönem 4

| | | |
|---|---|---|
| 2 | Evet | 50 |
| | Hayır | 1 |
| 3 | Evet | 36 |
| | Hayır | 15 |
| 4 | Evet | 4 |
| | Hayır | 46 |

5. Evet

卌 卌 卌 卌 卌
卌 卌 卌 卌 |

(46)

---

Hayır

||||

(4)

---

6. Evet

卌 卌 卌

(15)

---

Hayır

卌 卌 卌 卌
卌 卌 卌

(35)

---

7. Evet

卌 卌 卌 卌 卌
卌 卌 卌 卌 ||||

(49)

---

Hayır

|

(1)

---

8. Ağız içi tarayıcı.

(50)

卌 卌 卌 卌 卌
卌 卌 卌 卌 卌

Geleneksel Ölçü Yöntemi

(?) | (?)

9

Evet — 50

Hayır — 0

10

Ucuz — 7

Konfor — 46

Hızlı — 46

11.

Ses — 3

Manyetik — 6

Optik — 48

12.

TME — 9

isk. sınıf 2 — 24

Implant — 33

Protez — 49

Seffaf plak — 44

13.

Model (43)

Max gen (22)

Indirek bond (27)

Seffaf plak (46)

Myofonk (21)

Cevap vermeyen (1)

Scanned with CamScanner

# DONEM 5

| | Evet | Hayır |
|---|---|---|
| 2. | 52 | |
| 3. | 41 | 11 |
| 4. | 15 | 37 |
| 5. | 46 | 6 |
| 6. | 16 | 36 |
| 7. | 51 | 1 |

[illegible] B (8) BÖLÜM 5

| | |
|---|---|
| Ağız içi tarayıcı (49) | Geleneksel (3) |

| | |
|---|---|
| 5. Evet (48) | Hayır (4) |

| | | |
|---|---|---|
| 10. Uzun (16) | Hasta konfor (42) | Hızlı tedavi (37) |

| | | |
|---|---|---|
| 11. Ses (7) | Manyetik (10) | Optik (42) |

| | | | |
|---|---|---|---|
| 12 TME (15) | Sınıf 2 (34) | İmplant (33) | Protez (43) |

Şeffaf (39)

| | | | | |
|---|---|---|---|---|
| 13. Model (40) | Exp. (27) | İndirekt (37) | Şeffaf (44) | Myfonk. (22) |

# Ödem 5

| | Evet | Hayır |
|---|---|---|
| 2 | 44 | |
| 3 | 27 | 17 |
| 4 | 1 | 43 |
| 5 | 42 | 2 |
| 6 | 8 | 36 |
| 7 | 43 | 1 |

Scanned with CamScanner

# DÖNEM 5

**8**

| [illegible]anayıcı | Geleneksel |
|---|---|
| 42 | 2 |

**9**

| Evet | Hayır |
|---|---|
| 43 | 1 |

**10**

| Ucuz | Konfor | Hızlı |
|---|---|---|
| 6 | 37 | 31 |

**11**

| Ses | MR | Optik |
|---|---|---|
| 5 | 7 | 36 |

**12**

| PME | C12 | İmplant | Protez | Şeffaf Plak |
|---|---|---|---|---|
| 10 | 23 | 32 | 38 | 41 |

**13**

| Model | Eksp. | İndirekt | Şeffaf plak | Miyofonks. |
|---|---|---|---|---|
| 36 | 21 | 24 | 43 | 23 |
